# Core–Shell Polyaniline–Carbon Nanotube Electrodes with Engineered Interfaces for High-Performance Ionic Polymer–Gel Composite Actuators

**DOI:** 10.3390/gels12040270

**Published:** 2026-03-25

**Authors:** Jintao Zhao, Yang Cao, Zhenjie Zhang, Dongyu Yang, Mingchuan Jia

**Affiliations:** 1 Weihai Campus, Harbin University of Science and Technology, Weihai 264300, China; 2 School of Mechanical and Power Engineering, Harbin University of Science and Technology, Harbin 150080, China

**Keywords:** actuators, gel membrane, nafion, multi-walled carbon nanotubes, polyaniline, interfacial engineering, smart materials

## Abstract

Ionic polymer–metal composites consist of an ion-conducting polymer–gel membrane sandwiched between two flexible electrodes, representing a class of soft electroactive materials capable of large deformation under low voltage. The gel membrane, swollen with solvent, facilitates ion migration under an electric field, enabling actuation. Tailoring the interfacial architecture between the electrode and the polymer–gel membrane is pivotal for advancing high-performance IPMC actuators. This study presents a comparative investigation of three core–shell nanocomposite electrodes, fabricated via in situ polymerization, for IPMC applications. Among these, the polyaniline-coated multi-walled carbon nanotube composite exhibits a deliberately designed hierarchical structure, with a specific surface area of 32.345 m^2^·g^−1^ and a conductive doped polyaniline shell, as confirmed through XPS analysis. This optimized interface enables superior charge storage and transport, endowing the corresponding electrode with a specific capacitance of 40.28 mF·cm^−2^ at 100 mV·s^−1^—3.2 times greater than that of conventional silver-based electrodes—along with a reduced sheet resistance. When integrated with a Nafion ion–gel membrane, the PANI@MWCNT electrode achieves a 67% increase in force density and a larger displacement output compared to standard devices, directly correlated with its enhanced electrical and electrochemical properties. This work highlights the critical role of core–shell interfacial engineering in governing electromechanical performance at the electrode–gel interface and offers a practical design strategy for developing high-performance, cost-effective IPMC actuators for soft robotics, flexible electronics, and related applications.

## 1. Introduction

Ionic polymer–metal composites (IPMCs) are a class of soft electroactive materials characterized by high force-to-mass ratios, low operating voltages, and rapid current responses, making them promising candidates for soft robotics, flexible electronics, and biomedical actuators [1,2,3,4,5]. Traditional IPMCs typically employ noble metal electrodes coated onto ion-exchange membranes, such as Nafion, to enable efficient charge transport and actuation [6,7,8]. However, metallic electrodes often face limitations, including high cost, limited mechanical flexibility, and restricted tunability, motivating the exploration of polymer- and carbon-based electrode alternatives [9,10,11,12,13].

Conductive polymers, such as polypyrrole (PPy), polythiophene, and polyaniline (PANI), have been extensively investigated as flexible IPMC electrodes due to their tunable electrochemical properties, processability, and mechanical compliance [14,15,16,17,18,19,20]. Among these, PANI combined with multi-walled carbon nanotubes (MWCNTs) has been widely reported to enhance electrode conductivity and actuation performance [21,22,23,24,25]. Despite this, most prior studies focus on material composition and simple polymer–nanotube integration. Limited insight is available on how hierarchical core–shell architectures and electrode–gel interfacial engineering influence electromechanical performance [26,27,28,29]. In particular, the synergistic interaction between a conductive core and a redox-active PANI shell, forming a three-dimensional interpenetrating interface with the hydrated Nafion gel, has not been fully quantified in terms of energy storage and actuation efficiency.

To address these gaps, the present study systematically designs and evaluates core–shell nanocomposite electrodes for IPMC applications. The main novelties of this work are threefold:

(1) A rational interface engineering of the PANI@MWCNT core–shell structure, optimizing electron transport pathways and ion accessibility.
(2) An enhancement of electrode–gel interfacial contact, improving electromechanical transduction efficiency.
(3) The direct correlation of electrode structural features with electrochemical and actuation performance, enabling quantitative comparison and mechanistic insights across PANI@MWCNT, PPy@MWCNT, and PANI@FeC electrodes under identical fabrication and testing conditions.

By decoupling the contributions of the core material, polymer shell, and interfacial morphology, this study elucidates the critical role of core–shell architecture, interface engineering, and ion transport dynamics in improving IPMC performance. These findings provide mechanistic insights into electrode design strategies and highlight practical approaches for developing high-performance, cost-effective soft actuators for robotics, flexible electronics, and biomedical applications.

## 2. Results and Discussion

### 2.1. Polymer-Electrode IPMC Conductivity Test Analysis

The hot-pressing process elevates electrode resistance in acidic environments. Given the inherent conductivity limitations of polymeric materials relative to metals, a metal–polymer hybrid electrode architecture is proposed for enhanced surface conductivity.

Table 1 summarizes the resistance measurements of polymer-based IPMC electrodes. Notable performance variations occur among the three IPMC types: PANI@FeC and PPy@MWCNT exhibit a higher resistance, while PANI@MWCNT demonstrates lower values. This variation originates from two factors: the inherent resistivity of FeC compounds and interfacial effects in the core–shell structure. A positive correlation exists between the surface sheet resistance ratios. These variations arise from limitations in the room-temperature solidification process: the inadequate bonding between resin materials and core–shell polymers or ion-exchange membranes and polymer agglomeration during coating leading to void formation and electrode inhomogeneity.

### 2.2. Study on the Energy Storage Performance of Polymer-Electrode IPMC

**Figure 1:**
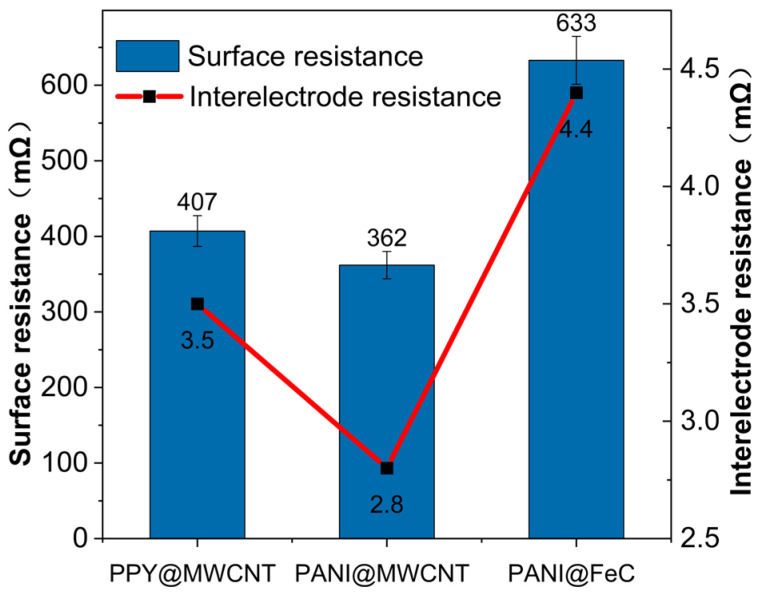
Surface resistance and sheet resistance of the three types of IPMC electrodes.

**Figure 2:**
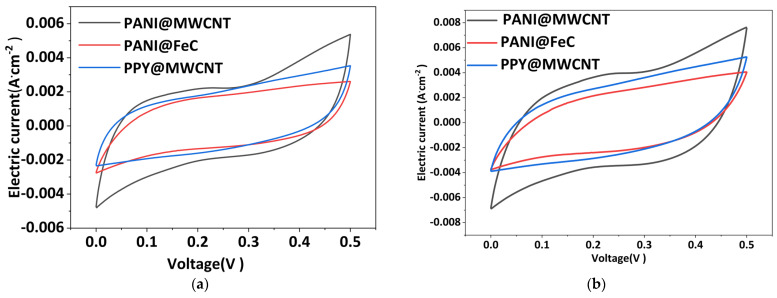

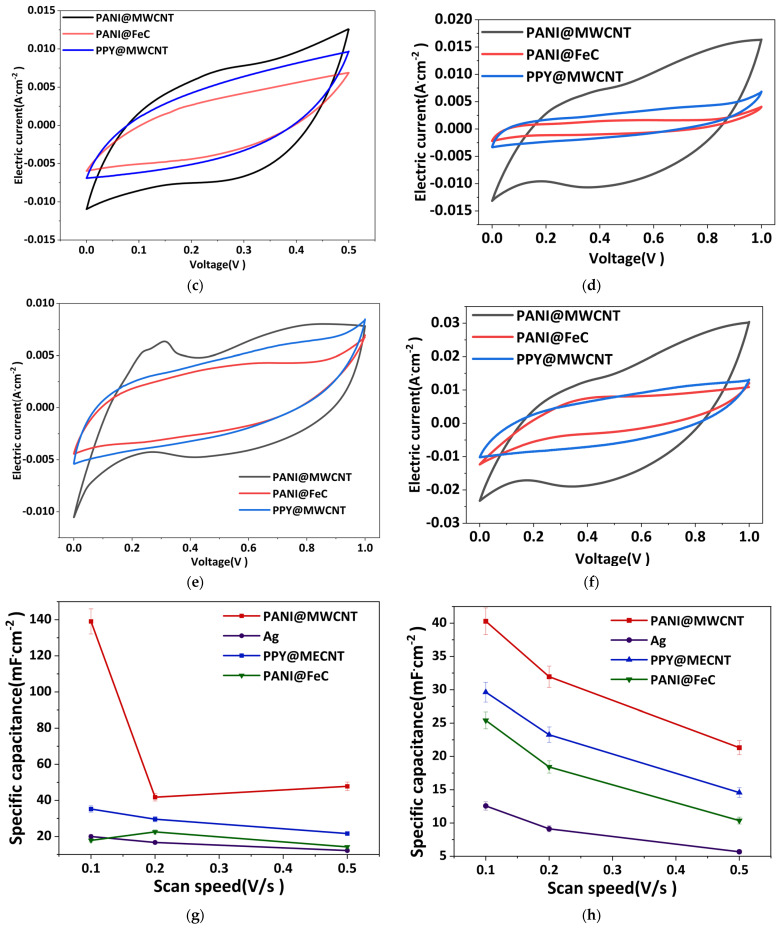
Cyclic voltammetry (CV) curves of polymer-based IPMC electrodes at different potential windows and scan rates: (**a**) 0–0.5 V, 100 mV·s^−1^; (**b**) 0–0.5 V, 200 mV·s^−1^; (**c**) 0–0.5 V, 500 mV·s^−1^; (**d**) 0–1.0 V, 100 mV·s^−1^; (**e**) 0–1.0 V, 200 mV·s^−1^; (**f**) 0–1.0 V, 500 mV·s^−1^; (**g**) comparison of specific capacitance at 0.5 V; (**h**) comparison of specific capacitance at 1.0 V.

The electrochemical performance of three polymer-based IPMCs was evaluated via cyclic voltammetry and compared with conventional silver-electrode IPMCs. Figure 1 and Figure 2 present the surface resistance, sheet resistance, and CV curves.

An analysis of the CV curves reveals that PANI@FeC and PPy@MWCNT electrodes demonstrate smooth, rectangular voltammograms, indicating stable electrochemical behavior with uniform charge/discharge characteristics and effective double-layer formation. Conversely, the PANI@MWCNT electrode exhibits pronounced fluctuations during potential scanning, particularly at wider potential windows. Specific capacitance comparisons at 0.5 V and 1.0 V are also presented in Figure 2g,h, highlighting the superior charge storage capability of PANI@MWCNT relative to the other polymer electrodes.

Five factors contribute to this phenomenon: first, a structural reorganization of the polyaniline wrapping layer during electrochemical processes; second, enhanced charge transport through conductive MWCNT networks; third, redox reactions of residual free polyaniline; fourth, interfacial charge transfer resistance at polymer–nanotube junctions; fifth, a non-uniform polyaniline coating thickness. These effects become more pronounced at wider potential windows due to electrolyte concentration gradients and surface oxidation processes. Specific capacitance analysis reveals distinct scanning-rate dependence: PANI@MWCNT shows an unstable performance at wider potential windows but an improved stability at narrower ranges. Two primary mechanisms explain this behavior: adsorption–desorption kinetics at lower scan rates and interfacial impedance effects at higher current densities. While the capacitance initially increases with yjr scan rate due to enhanced charge transfer, it eventually saturates as ion transport becomes rate-limiting.

This study systematically evaluates three polymer-electrode IPMCs, demonstrating their enhanced capacitance compared to conventional silver-electrode IPMCs. All three polymer-based electrodes exhibit a significantly improved capacitance, indicating a superior energy storage capacity relative to silver electrodes. This contrast confirms the material-dependent nature of energy storage performance. The core–shell architecture of these polymeric materials features a stratified structure, which according to prior research enhances the electrode storage capability through three mechanisms:

First, the core–shell morphology substantially increases the effective surface area, providing more active sites for charge storage. Second, the conductive shell layer optimizes electron transport, reducing the overall electrode resistance. Third, a distributed double-layer effect forms at core–shell interfaces, where the polymer shell functions as a charge storage unit while the core establishes potential gradients to facilitate charge accumulation.

The superior electrochemical performance of the PANI@MWCNT electrode can be attributed to its well-defined core–shell architecture, which provides a high specific surface area and a porous network conducive to ion diffusion. The presence of micropores facilitates rapid electrolyte penetration and charge accommodation, while the conductive PANI shell in its emeraldine salt form ensures efficient electron transport. In contrast, the PANI@FeC electrode suffers from limited interfacial contact and a lower intrinsic conductivity of the FeC core, leading to a reduced capacitance and slower charge transfer kinetics. The PPy@MWCNT electrode, although exhibiting stable capacitive behavior, lacks the redox-active nitrogen species present in PANI, which limits its pseudocapacitive contribution. These findings underscore the importance of both material selection and interfacial engineering in determining the overall energy storage and actuation performance of IPMC devices.

The capacitive behavior observed in CV curves reflects not only the intrinsic properties of the electrode materials but also the ion transport dynamics within the hydrated Nafion gel layer. The superior performance of PANI@MWCNT can be attributed to its hierarchical structure, which provides efficient pathways for ions migrating from the gel bulk to the electrode surface. The electrode–gel interface serves as the primary site for charge accumulation, and its optimization through core–shell engineering directly enhances specific capacitance.

**Figure 3:**
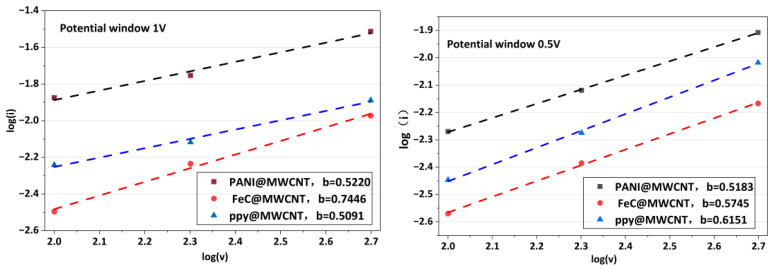
B-value analysis.

To further elucidate the energy storage kinetics of the electrodes, the electrochemical processes were analyzed by combining b-value analysis, Dunn’s method, and EIS fitting. As shown in Figure 3, the calculated b-values of the three electrodes under different potential windows were all between 0.5 and 1.0, indicating that their charge storage behavior was governed by the combined effects of ion diffusion and surface-controlled pseudocapacitive processes. Furthermore, the quantitative separation of diffusion-controlled and capacitive contributions is summarized in Table 1. The results show that the capacitive contribution increased with an increasing scan rate, whereas PANI@MWCNT exhibited a relatively stable diffusion-dominated characteristic under different potential windows.

**Figure 4:**
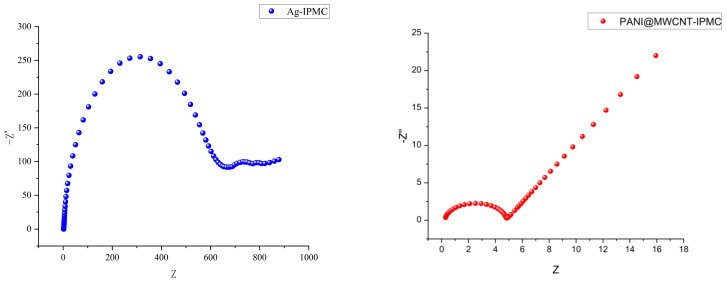
Nyquist comparison chart.

**Figure 5:**
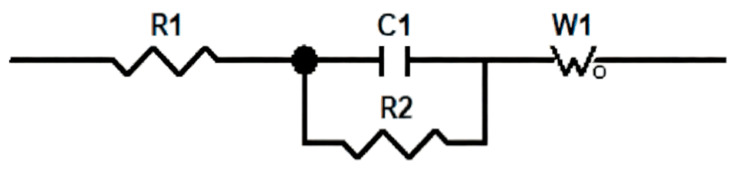
Equivalent circuit diagram.

To evaluate the electrochemical performance of the fabricated IPMC, cyclic voltammetry measurements were first conducted for all electrode types, allowing a preliminary comparison of their charge storage capabilities. Based on these CV results, the PANI@MWCNT electrode was identified as the optimal material. Subsequently, electrochemical impedance spectroscopy (EIS) was performed on the PANI@MWCNT-IPMC electrode. Combined with the equivalent-circuit fitting results in Figure 4 and Figure 5 and in Table 2, the Nyquist plot of PANI@MWCNT-IPMC displayed a smaller semicircle diameter in the high-frequency region and a more pronounced diffusion-related response in the low-frequency region than that of the conventional silver-electrode IPMC; the PANI@MWCNT electrode possesses a lower interfacial charge transfer resistance and more efficient ion transport capability. Collectively, these results demonstrate that the PANI-coated multi-walled carbon nanotube architecture effectively improves the electrochemical kinetics at the electrode–gel interface, thereby enhancing both the charge storage capability and the actuation performance of the IPMC.

### 2.3. Study on the Impact of IPMC Energy Storage Performance on Drive Performance of Polymer Electrodes

#### 2.3.1. Polymer-Electrode IPMC Displacement Performance Study

**Figure 6:**
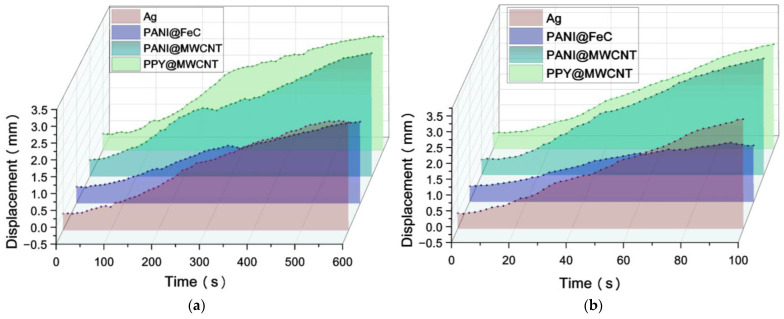
Polymer-electrode IPMC displacement test diagram: (**a**) 3 V displacement diagram; (**b**) 5 V displacement diagram.

To investigate the correlation between the energy storage capacity and actuation performance, three polymer-electrode IPMCs with varying capacitance characteristics were evaluated alongside conventional silver-electrode IPMCs. The actuation performance was assessed under 3 V and 5 V DC excitation using laser displacement sensors (7 mm working distance) as shown in Figure 6.

Comparative testing indicates that PANI@FeC demonstrates inferior actuation, while PPy@MWCNT and PANI@MWCNT demonstrate a comparable enhanced performance. This performance variation reflects differences in core–shell structural integrity and capacitive behavior. The higher capacitance of PPy@MWCNT and PANI@MWCNT electrodes enables a more efficient electromechanical transduction.

Displacement characterization confirms two key dependencies: (a) the strong material dependence of actuation performance, particularly core–shell architecture effects, and (b) a positive correlation with energy storage capacity until saturation. The observed rate modulation, with a slower initial response at a higher capacitance transitioning to an accelerated displacement, suggests dynamic ion redistribution processes. The actuation mechanism of IPMC involves coupled processes: ion migration through the hydrated gel membrane, charge accumulation at the electrode–gel interface, and mechanical deformation of the polymer–gel network. The enhanced displacement of PANI@MWCNT-based IPMC demonstrates that optimized interfacial engineering improves all three steps. The gel membrane acts both as an ion reservoir and a mechanical matrix, and its coupling with the nanostructured electrode determines the overall actuation efficiency.

#### 2.3.2. Mechanical Characterization of IPMC for Polymer Electrodes

**Figure 7:**
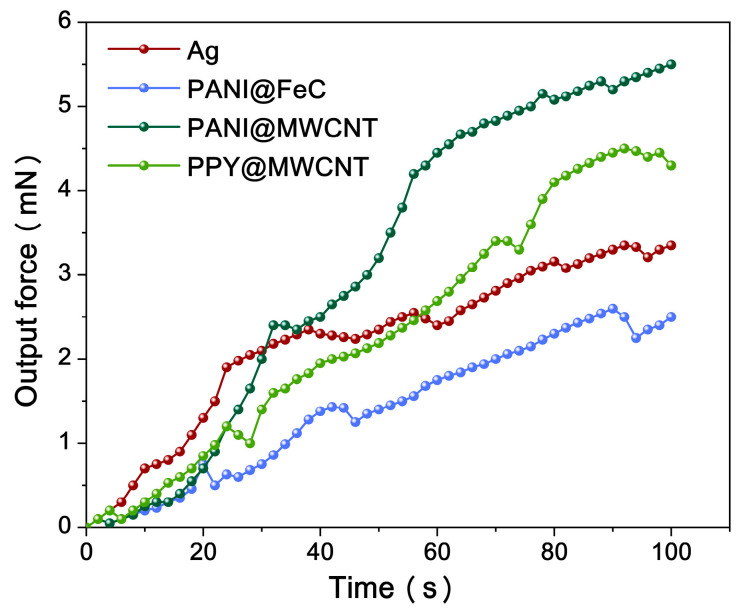
Output force responses.

The mechanical response of the IPMC is characterized in Figure 7 using the parameters listed in Table 3. The operational benchmark was defined as 90% of the maximum output force, representing sustained peak performance conditions. The experimental results confirm that PANI@FeC IPMC exhibits an inferior actuation performance compared to PANI@MWCNT and PPy@MWCNT variants, attributable to its wave-absorbent properties that dissipate charge during operation.

Figure 7 demonstrates the superior mechanical response of PANI@MWCNT IPMC, exhibiting continuous force enhancement beyond 100 s of operation without rebound—indicating ongoing ion migration processes prior to dynamic equilibrium establishment. The correlation with capacitance measurements confirms that a larger capacitance directly affects the IPMC’s ultimate output force.

**Figure 8:**
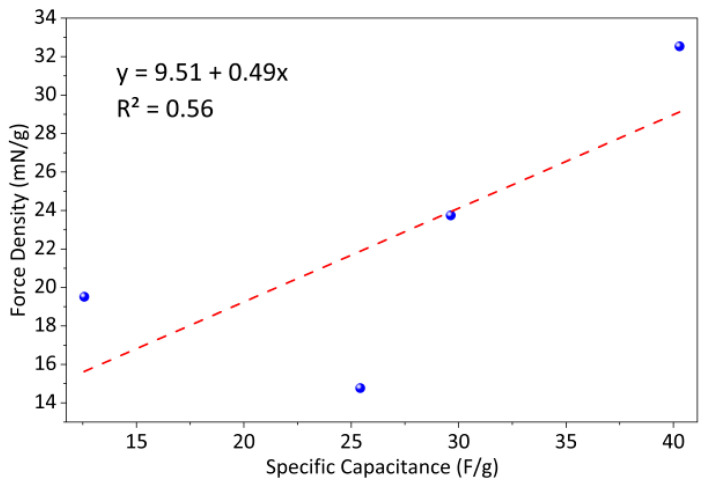
Capacitance–force density fitting.

As shown in Figure 8, the force density of polymer-electrode IPMCs increases linearly with specific capacitance, with each additional 1 mF·cm^−2^ corresponding to an increase of approximately 0.49 mN·g^−1^. This quantitative relationship indicates that electrodes with a higher charge storage capacity accumulate a greater charge at the electrode–gel interface, generating stronger local electric fields. These fields drive cation migration within the hydrated Nafion membrane, producing asymmetric swelling of the polymer network. The resulting bending moment enhances both the tip displacement and force density of the actuator. The improved actuation performance of the PANI@MWCNT IPMC thus arises from the synergistic effect of its hierarchical core–shell electrode architecture and increased specific capacitance. This mechanism, linking electrochemical charge accumulation to mechanical deformation, is consistent with recent studies on IPMC actuators [30], where cation migration and localized swelling under applied voltage are identified as the primary drivers of bending deformation. The force output is directly related to the amount of charge stored at the electrode–gel interface and the efficiency of ion transport through the gel network. The PANI@MWCNT electrode’s porous architecture enables intimate contact with the hydrated Nafion gel, maximizing the active interfacial area for charge accumulation.

The force density of polymer-electrode IPMCs increases with the specific capacitance. The PANI@MWCNT electrode with a hierarchical porous structure achieves the highest capacitance of 40.28 ± 1.2 millifarads per square centimeter and a maximum force density of 32.54 ± 0.6 millinewtons per gram (*n* = 3), representing a 67 percent increase over silver electrodes. Linear fitting shows that each additional millifarad per square centimeter enhances the force density by 0.49 millinewtons per gram. The improvement is attributed to the optimized electrode–gel interface, which promotes rapid ion migration and a higher charge accumulation, generating a stronger internal electric field and increased mechanical output.

To highlight the application-specific performance of MWCNT electrodes in IPMC, a comparative summary is presented in Table 4. The table demonstrates that the PANI@MWCNT electrode developed in this study achieves a superior specific capacitance, force density, and displacement output relative to other MWCNT- and polymer-based electrodes reported in the literature. These enhancements are attributed to the hierarchical core–shell architecture, which optimizes the electrode–gel interface and facilitates ion penetration through a microporous network. The results clearly underscore the novelty and practical advantages of the PANI@MWCNT design in soft actuator applications.

### 2.4. Structural and Chemical Characterization

**Figure 9:**
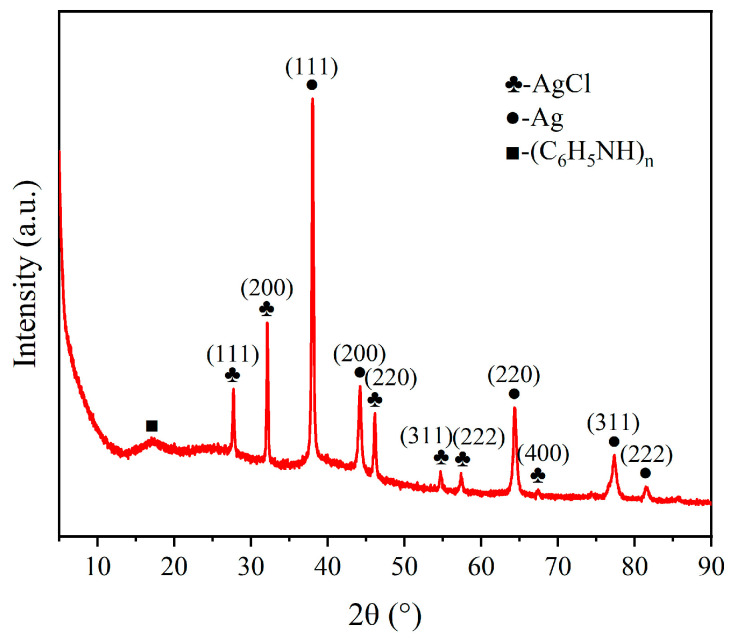
XRD spectrum.

As shown in Figure 9, the XRD spectrum shows that the sample contains the Ag/AgCl crystalline phase, namely the main diffraction peaks: 38.1° Ag(111), 32.2° AgCl(111), etc. As shown in the XRD pattern, the diffraction peaks assigned to Ag/AgCl were observed, which mainly originated from the silver adhesive used during electrode preparation. In the present system, the silver adhesive functioned primarily as a conductive binder, improving the adhesion of the composite coating to the Nafion membrane and reducing contact resistance within the electrode layer. The partial formation of Ag/AgCl may occur during processing or electrochemical testing in acidic or chloride-containing environments. Although the silver-containing binder may provide a certain background contribution to the overall conductivity and electrochemical response, the markedly improved capacitance and actuation performance of the PANI@MWCNT electrode compared with the conventional silver-electrode IPMC suggest that the observed enhancement cannot be explained by the binder alone. Instead, it is more reasonably associated with the engineered core–shell architecture and the improved electrode–gel interfacial properties of the PANI@MWCNT electrode. At the same time, the broad and gentle diffraction pattern in the 20–30° region confirms the existence of amorphous PANI.

**Figure 10:**
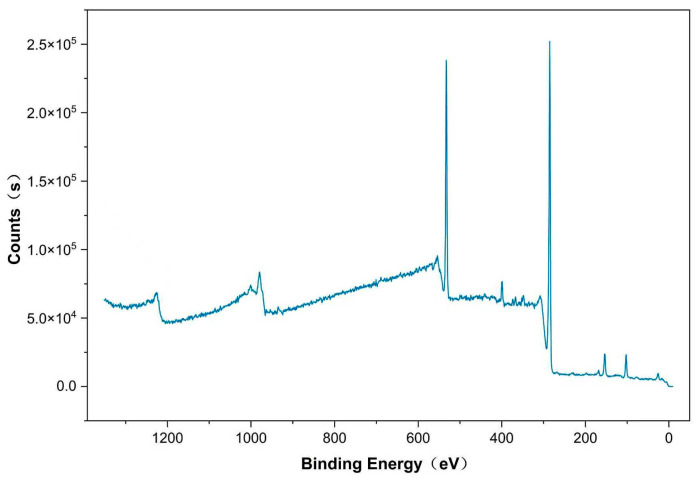
XPS survey.

**Figure 11:**
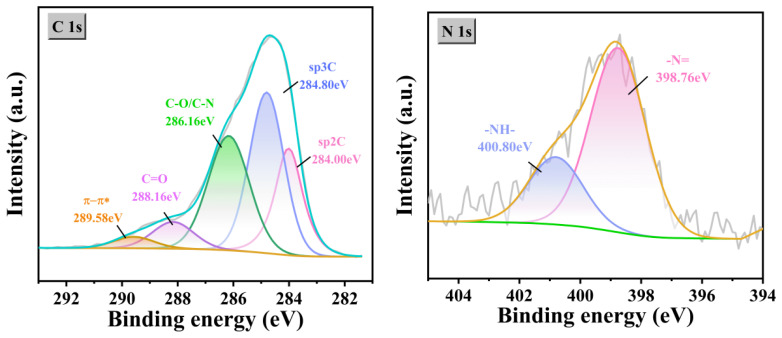
High-resolution spectrum, * denotes the antibonding state in the π–π transition.

In Figure 10 and Figure 11, the XPS data clearly confirm that the sample is a polyaniline-coated multi-walled carbon nanotube. The high-resolution N 1s spectrum can be deconvoluted into three components corresponding to the quinoid imine (=N–), benzenoid amine (–NH–), and positively charged nitrogen (N^+^). The significant presence of the N^+^ component confirms that PANI is in the conductive emeraldine salt (doped) state. The C 1s spectrum shows characteristic peaks for C–C/C=C (sp^2^ carbon from MWCNTs), C–N (from PANI), and C=O, confirming the coexistence of both components and effective composite formation.

The voltammetric response originates from the synergistic interaction between two types of surface-oxidized structures, oxygen-containing functional groups within the carbon skeleton and oxidized nitrogen species in polyaniline, collectively constituting the primary origin of the electrochemical activity.

**Figure 12:**
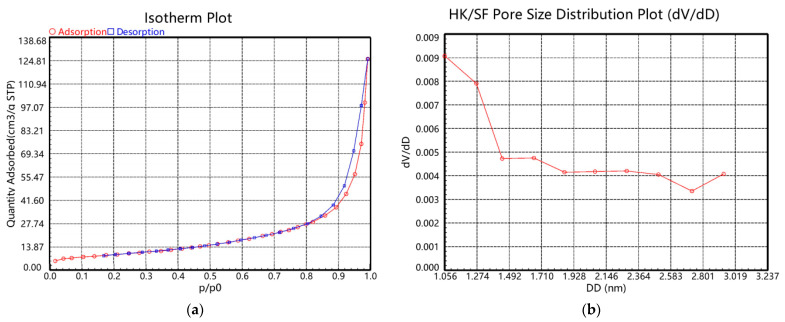
Porous structure characterization of the PANI@MWCNT composite: (**a**) isotherm plot and (**b**) pore size distribution plot.

As shown in Figure 12, based on nitrogen adsorption–desorption measurements, the PANI@MWCNT composite exhibits a specific surface area of 32.345 m^2^·g^−1^ and a total pore volume of 0.189 cm^3^·g^−1^. Significantly, the material demonstrates a pronounced microporosity (0.014 cm^3^·g^−1^) with a most probable pore size of 1.273 nm, confirming the successful incorporation of micropores by polyaniline and its effective coating on the carbon nanotube surfaces, the detailed BET surface characteristics are summarized in Table 5. The hierarchical porous structure of PANI@MWCNT is particularly advantageous for electrode–gel interfacial contact. The micropores facilitate the penetration of the hydrated Nafion gel network into the electrode layer, creating a 3D interpenetrating interface that enhances ion accessibility and reduces charge transfer resistance. This intimate contact between the electrode and the polymer–gel membrane is critical for efficient electromechanical transduction.

**Figure 13:**
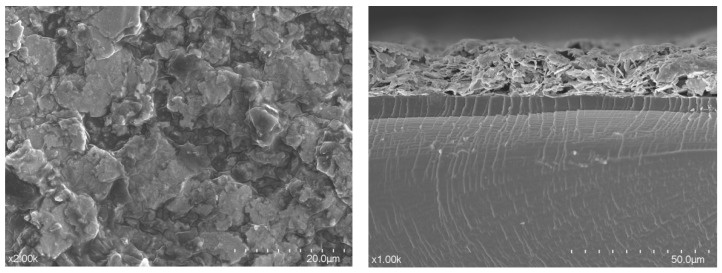
SEM micrograph.

The SEM images of PANI@MWCNT-IPMC are shown in Figure 13. The surface image (×2000, 20 μm) reveals a uniform coverage of PANI-coated MWCNTs. The cross-sectional image (×1000, 50 μm) shows the electrode thickness and stacked layered structure on the Nafion membrane, confirming dense, well-adhered electrodes that support efficient ion transport and enhanced actuation performance. Energy-dispersive X-ray spectroscopy (EDS) mapping of the PANI@MWCNT electrodes further confirmed a uniform distribution of nitrogen within the PANI shell, indicating a complete coverage over the MWCNT cores. Carbon was the dominant element, consistent with the polymer backbone, while localized silver signals correspond to the underlying conductive electrode layer. These results collectively support the formation of a continuous, dense, and conductive interface layer, which enhances ion transport and contributes to the improved actuation performance of the IPMC.

**Figure 14:**
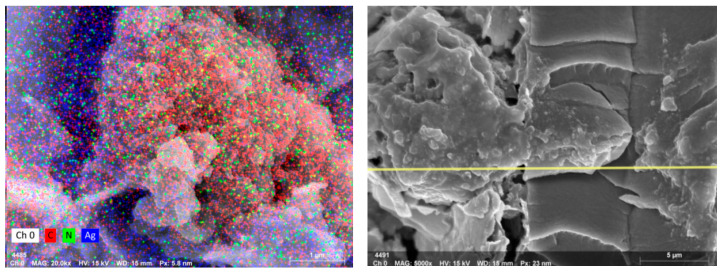
EDS micrograph, the yellow line indicates the EDS line-scan path.

Additionally, as shown in Figure 14 the high-magnification SEM images indicate that the PANI coating forms a conformal shell over the MWCNT cores without obvious aggregation, maintaining a high surface area. The cross-sectional images show a consistent electrode thickness across the membrane, suggesting a uniform deposition and good reproducibility. While the exact internal pore network and ion migration pathways cannot be directly visualized from SEM alone, the observed porous morphology of the PANI shell and the well-adhered layered structure qualitatively suggest an enhanced ionic accessibility and more efficient charge transport within the IPMC, supporting the observed improvements in electromechanical performance.

### 2.5. Research on the Influence of Humidity and Temperature on the Output Displacement of Polymer-Electrode IPMC

**Figure 15:**
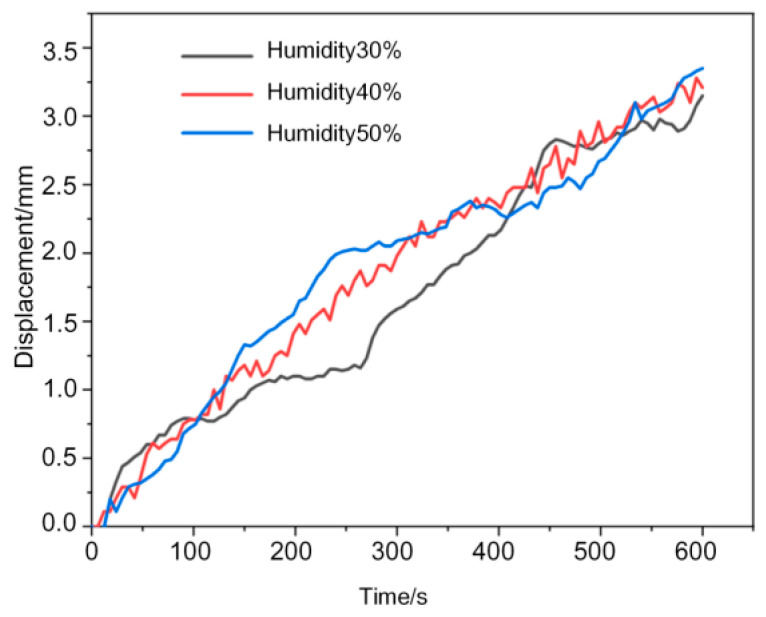
Humidity impact curve.

**Figure 16:**
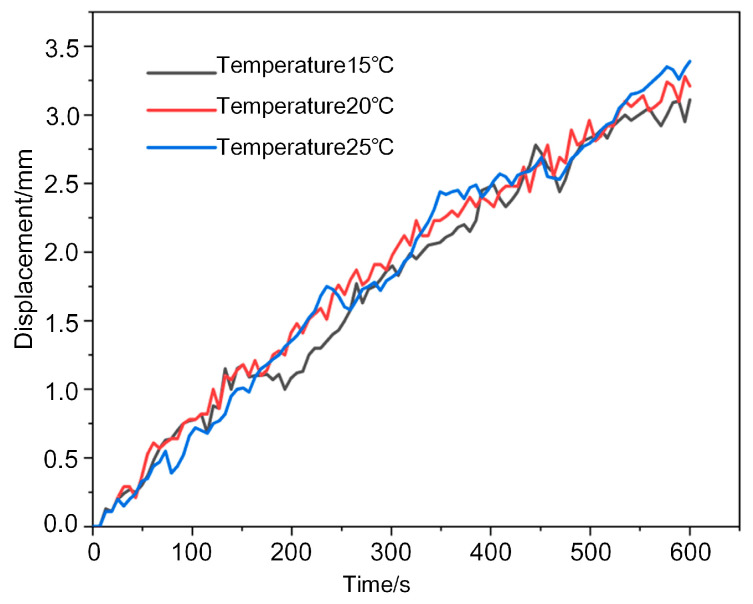
Temperature effect curve.

The effects of ambient humidity and temperature on the actuation performance of PANI@MWCNT-IPMC were evaluated because both factors may influence membrane hydration and ion mobility. As shown in Figure 15 under relative humidities of 30%, 40%, and 50%, the maximum displacement increased slightly from 3.15 to 3.35 mm. This change was within the normal fluctuation range of the actuator, indicating a limited effect of humidity under the tested conditions. Similarly, as shown in Figure 16 increasing the temperature from 15 to 25 °C led to only a slight increase in the maximum displacement. The weak dependence on humidity and temperature is attributed to the relatively thick electrode layer, limited membrane permeability, and restricted ion mobility in the commercial Nafion membrane. These results indicate that, under normal indoor conditions, the actuation performance is governed mainly by the electrode–gel interface and ion transport within the membrane rather than by moderate environmental variations.

## 3. Conclusions

This study demonstrates that the interfacial architecture of core–shell nanocomposites critically governs the performance of ionic polymer–metal composite actuators. The optimized polyaniline-coated carbon nanotube electrode, with its hierarchical structure and high surface area, exhibits a specific capacitance 3.2 times greater than conventional silver electrodes and the lowest sheet resistance. When integrated with a Nafion ion–gel membrane, the PANI@MWCNT electrode achieves intimate electrode–gel interfacial contact, enabling efficient charge storage and transport. Consequently, the corresponding actuator achieves a 67% increase in force density and an enhanced displacement. These results underscore core–shell design as a decisive strategy for engineering high-performance electroactive polymer composites through interfacial control between nanostructured electrodes and polymer–gel membranes. The findings provide design principles applicable to a range of gel-based soft devices, including soft robotics, biomedical actuators, and flexible electronics.

## 4. Materials and Methods

All chemical reagents were used as received without additional purification. Nafion D520 solution (5 wt%, DuPont, Wilmington, DE, USA) served as the ion-exchange matrix. Multi-walled carbon nanotubes (MWCNT, >95% purity, outer diameter 20–30 nm) were sourced from Shenzhen Hongdachang Jinhua Technology Co., Ltd. (Shenzhen, China). Zinc oxide nanoparticles (50 nm average size) and other reagents including hydrochloric acid (0.1 M), methyl orange (ACS grade), ammonium persulfate (analytical grade), aniline (redistilled), pyrrole (distilled), and ferric chloride (anhydrous) were obtained from Sinopharm Chemical Reagent Co. (Shanghai, China). Silver foil (99.9% purity) and dimethyl sulfoxide (HPLC grade) were supplied by Nanjing Material Technology Co., Ltd. (Nanjing, China) and Xilong Scientific Co., Ltd. (Guangzhou, China), respectively.

### 4.1. Material Preparation

The Nafion D520 membrane was pretreated prior to electrode coating by boiling in 3% H_2_O_2_ solution for 1 h to remove organic contaminants, rinsing in deionized water for 1 h to remove residual peroxide, and then treating in 0.5 M H_2_SO_4_ at 80 °C for 1 h to convert it to the protonic form. The membrane was thoroughly rinsed with deionized water to remove excess acid, ensuring a clean and protonated surface to facilitate uniform electrode deposition and optimal IPMC actuation performance.

**Figure 17:**
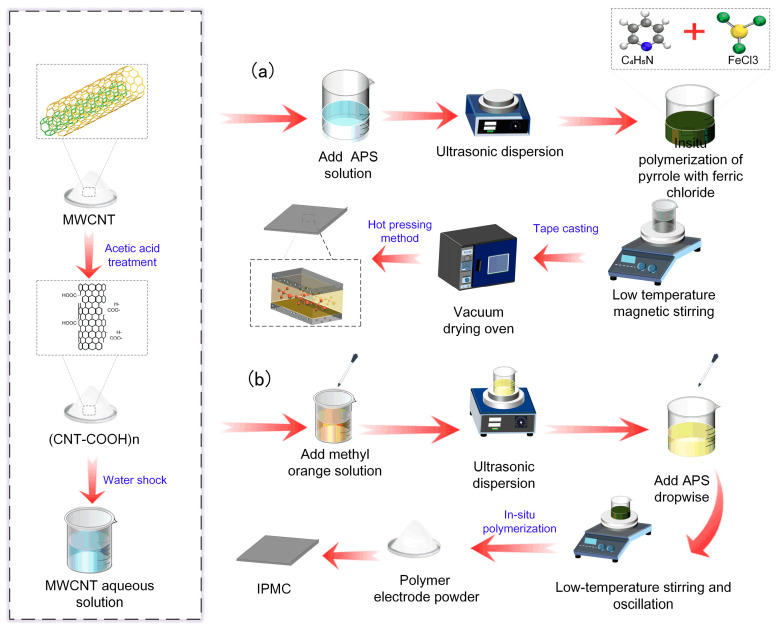
Preparation process of polymer composite electrode IPMC: (**a**) Preparation process of PANI-coated MWCNT IPMC. (**b**) Preparation process of PPy-coated MWCNT IPMC.

As shown in Figure 17, multi-walled carbon nanotubes (MWCNTs, 100 mg) were acid-treated in 0.5 M HCl and ultrasonicated for 30 min to achieve uniform dispersion. Aniline monomer (196 μL, 0.1 M) was dissolved in 40 mL of 0.5 M HCl, and ammonium persulfate (APS) was dissolved in 10 mL of 0.5 M HCl to give a 1:1 molar ratio of aniline to APS. The solutions were mixed at 0–5 °C and stirred continuously for 6 h, producing a uniform PANI coating on the MWCNTs. The precipitate was collected through filtration, washed with ethanol and deionized water, dried under vacuum at 60 °C for 12 h, and ground to a fine powder.

MWCNTs were also coated with polypyrrole (PPy) using a similar procedure in Figure 17. Acid-treated MWCNTs (100 mg) were dispersed in 0.1 M HCl through ultrasonication for 30 min. Pyrrole monomer was polymerized on the MWCNT surface using APS under continuous stirring at 0–5 °C for 6 h. One percent methyl orange solution was added to facilitate polymerization. The product was filtered, washed, dried under vacuum at 60 °C, and ground to powder.

**Figure 18:**
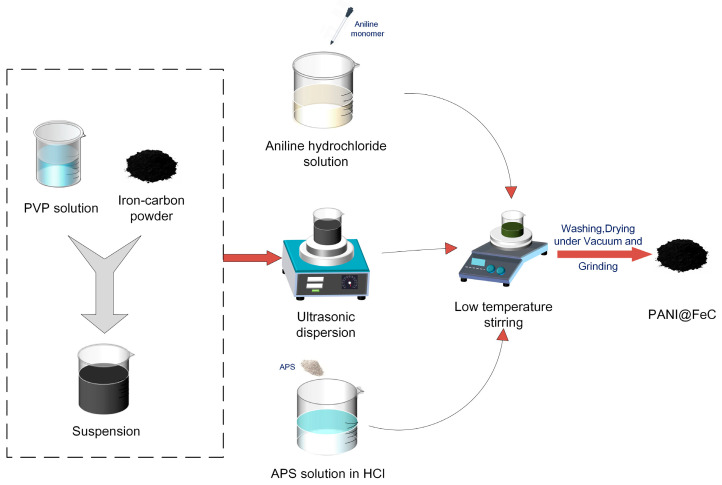
Preparation process of PANI@FeC composite.

As shown in Figure 18, The polyaniline-coated iron–carbon (PANI@FeC) composite was prepared by dispersing 200 mg of iron–carbon powder in 50 mL deionized water containing 100 mg polyvinylpyrrolidone (PVP) and ultrasonicating it for 30 min. Aniline (196 μL, 0.1 M) was dissolved in 40 mL 0.5 M HCl, and APS at the same molar concentration was dissolved in 10 mL 0.5 M HCl. The three solutions were mixed at 0 °C and stirred for 6 h to yield a green precipitate. The product was washed with ethanol and deionized water, dried under vacuum at 60 °C, and ground to powder.

The composite powder was dispersed in a mixed solvent of ethanol and N-methylpyrrolidone, followed by the addition of silver adhesive as a conductive binder. The silver adhesive was introduced to improve the adhesion between the composite coating and the Nafion membrane, enhance the mechanical integrity of the electrode layer, and reduce interparticle and contact resistance within the coating. The resulting slurry was magnetically stirred at 50 °C for 3 h to obtain a homogeneous dispersion before coating. The slurry was coated onto the pretreated Nafion membrane, allowed to rest to release trapped air, and pre-dried at 50–60 °C to produce a uniform, defect-free electrode layer. The resulting electrode layer loading was measured to be approximately 1.5 mg/cm^2^. The thickness of the fabricated IPMC electrodes was measured at multiple positions, yielding values between 0.173 mm and 0.343 mm, with an average thickness of 0.261 mm. The coated membrane was further pre-dried at 60 °C for 2 h, cured at 80 °C for 10 h, and finally hot-pressed at 80 °C for 10 h to enhance interfacial bonding. The resulting IPMC films were washed with warm water and demolded, ready for electrochemical and mechanical characterization.

### 4.2. Test Methods

The present work systematically characterizes the electrical and electrochemical properties of polymer electrodes. The electrical properties of the IPMC electrode layers were characterized in terms of surface resistance and sheet resistance. Because the electrode layers were thin and highly conductive, the measured values mainly reflected the sheet resistance characteristics of the coatings. Sheet resistance was measured using a four-point probe system to ensure measurement accuracy.

**Figure 19:**
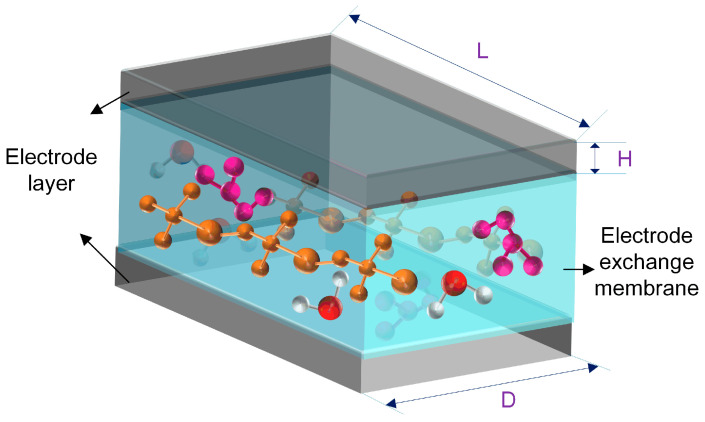
Schematic diagram of IPMC electrode.

Following the schematic in Figure 19, when the sample width *D* equals its length *L*, the sheet resistance R_0_ of the electrode layer can be calculated using Equation (1). In this equation, *R* (the numerator) represents the measured resistance of the electrode layer, *D* and *L* denote the sample width and length, respectively, in millimeters (mm), and *H* is the thickness of the electrode layer in millimeters (mm). This allows an accurate determination of the intrinsic sheet resistance of the electrode coating, excluding the contribution from the non-conductive Nafion membrane.

(1) $$R_{0}=\frac{\rho }{H}$$

IPMC actuation mechanisms depend on the interelectrode electric field, directly correlating with charge storage capacity. Consequently, supercapacitor characterization methods [31,32] including cyclic voltammetry [33], AC impedance spectroscopy, and galvanostatic charge/discharge techniques were adapted for performance assessment. Cyclic voltammetry was specifically selected for IPMC specific capacitance analysis [34].

The sheet resistance of the PANI@MWCNT electrode layer was measured using a four-probe system. In Equation (1), H refers specifically to the thickness of the electrode layer itself, excluding the non-conductive Nafion membrane. This allows quantification of the intrinsic electrical properties of the electrode material. While the complete IPMC assembly includes the Nafion membrane, measuring the surface resistance of the electrode alone provides a direct assessment of its conductivity and uniformity, which is critical for understanding and optimizing charge transport at the electrode–gel interface.

The actuation performance of the IPMC strips, 20 mm × 5 mm, was measured using a laser displacement sensor Keyence LK-G80, Japan, at a sampling rate of 1 kHz. DC voltages of 3 V and 5 V were applied via a programmable power supply, and tip displacement was recorded over 120 s. Force output was measured using a high-sensitivity load cell Transducer Techniques GSO-10, USA, positioned at the free end of the actuator. All tests were conducted under controlled ambient conditions at 25 °C and 40% relative humidity.

### 4.3. Electrochemical Characterization

The electrochemical performance of the PANI@MWCNT electrodes was evaluated using a CHI660E electrochemical workstation, Chenhua Instruments, in a conventional three-electrode setup, with platinum foil and Ag/AgCl serving as counter and reference electrodes, respectively.

Cyclic voltammetry, CV, measurements were conducted at scan rates of 100, 200, and 500 mV·s^−1^ within potential windows of 0.5 V and 1.0 V in 1 M H_2_SO_4_ electrolyte. The specific capacitance of the electrode was calculated from the CV curves using the following equation:

(2) $$C\;=\;\frac{1}{\mathrm{Av}\Delta V}\int_{\mathrm{Vmin}}^{\mathrm{Vmax}}i(V)\;\mathrm{dV}$$

where C is the specific capacitance in millifarads per square centimeter, i(V) is the current at voltage V, A is the electrode area in square centimeters, ν is the scan rate in volts per second, and ΔV = V_max_ − V_min_ is the potential window, with V_min_ and V_max_ denoting the lower and upper potential limits, respectively.

Electrochemical impedance spectroscopy, EIS, was performed in the frequency range of 10 mHz–100 kHz with an AC amplitude of 5 mV. EIS measurements were first conducted for IPMC electrodes at room temperature to provide an initial comparison of interfacial charge transfer resistance and ion transport characteristics, allowing an identification of the optimal electrode material for detailed analysis. All measurements were performed on three independent specimens (n = 3). The results are reported as mean ± standard deviation to reflect experimental variability.

## Figures and Tables

**Table 1:** Summary of b value analysis data.

Materials	Potential Window (V)	100 mV·s^−1^	200 mV·s^−1^	500 mV·s^−1^
PANI@MWCNT	0.5	21.65%	26.99%	38.78%
FeC@MWCNT	0.5	11.24%	15.22%	25.10%
PPy@MWCNT	0.5	21.41%	26.62%	39.36%
PANI@MWCNT	1	11.21%	35.71%	31.50%
FeC@MWCNT	1	58.55%	51.58%	71.00%
PPy@MWCNT	1	21.40%	29.36%	41.23%

**Table 2:** Fitting results.

Element	Freedom	Value	Error	Error%
Rs	Free(+)	0.26956	0.0057313	2.1262
C1	Free(+)	4.3222 × 10^−6^	3.1002 × 10^−8^	0.71727
Rct	Free(+)	4.5	0.021856	0.48569
W1-R	Free(+)	374.1	37.575	10.044
W1-T	Free(+)	35.77	5.1961	14.526
W1-P	Free(+)	0.70092	0.0030148	0.43012

**Table 3:** Output force test.

IPMC Classification	Maximum Output Force (mN)	Mass of Sample (g)	Force Density (mN·g^−1^)	First Arrival Time (s)	Duration (s)
Silver-Electrode IPMC	3.35	0.1716	19.52 ± 0.28	76	24
PANI@FeC IPMC	2.6	0.176	14.77 ± 0.29	82	20
PANI@MWCNT IPMC	5.5	0.169	32.54 ± 0.60	74	26
PPy@MWCNT IPMC	4.5	0.1818	23.75± 0.27	80	21

**Table 4:** Application status table of MWCNT in specific application scenarios.

Electrode Type	Core/Shell Material	Electrode Structure	Specific Capacitance (mF·cm^−2^)	Force Density (mN·g^−1^)	Key Innovations/Advantages
PANI@MWCNT (This Work)	MWCNT/PANI	Hierarchical core–shell porous	40.28	32.54	Optimized electrode–gel interface; microporous structure enhances ion accessibility; 3.2× capacitance; 67% force density improvement
PPy@MWCNT (This Work)	MWCNT/PPy	Core–shell	28.5	23.75	Stable electrochemical performance; lacks redox-active N species
PANI@FeC (This Work)	FeC/PANI	Core–shell	18.2	14.77	Limited interfacial contact; lower conductivity
PEDOT/MWCNT [10]	MWCNT/PEDOT	Core–shell	25.0	20.1	High flexibility and repeatability
Ag/Cu-PANI [22]	Ag/Cu/PANI	Composite	22.0	18.5	High conductivity; high cost
Conventional Ag Electrode (This Work)	Ag	Film	12.6	19.52	Standard reference

**Table 5:** Analysis results of BET surface characteristics.

Electrode Type	BET Surface Area (m^2^·g^−1^)	Total Pore Volume (cm^3^·g^−1^)	Average Pore Diameter (nm)
PANI@MWCNT-IPMC	45.2	0.12	10.5

## Data Availability

The original contributions presented in this study are included in the article. Further inquiries can be directed to the corresponding author.

## References

[1] Khaldi R.A., Maziz A., Alici G., Spinks G.M., Jager E.W. (2016). Bottom-up microfabrication process for individually controlled conjugated polymer actuators. Sens. Actuators B Chem..

[2] Bian C.S., Zhu Z.C., Bai W.F., Chen H., Li Y. (2020). Fast actuation properties of several typical IL-based ionic electro-active polymers under high impulse voltage. Smart Mater. Struct..

[3] He Q., Song L., Yu M., Dai Z. (2015). Fabrication, characteristics and electrical model of an ionic polymer metal-carbon nanotube composite. Smart Mater. Struct..

[4] Shen Q., Olsen Z., Stalbaum T., Trabia S., Lee J., Hunt R., Kim K.J., Kim J., Oh I.-K. (2020). Basic design of a biomimetic underwater soft robot with switchable swimming modes and programmable artificial muscles. Smart Mater. Struct..

[5] Shahinpoor M., Kim K.J. (2001). Ionic polymer-metal composites: I. Fundamentals. Smart Mater. Struct..

[6] Wang H.S., Cho J., Song D.S., Jang J.H., Jho J.Y., Park J.H. (2017). High-performance electro active polymer actuators based on ultrathick ionic polymer-metal composites with nanodispersed metal electrodes. ACS Appl. Mater. Interfaces.

[7] Byun J.M., Hwang T., Kim K.J. (2019). Formation of a gold nanoparticle layer for the electrodes of ionic polymer-metal composites by electroless deposition process. Appl. Surf. Sci..

[8] Yang L., Zhang D., Zhang X., Tian A., Hui X., Yang J. (2020). Fabrication of Cu/nafion-based ionic polymer metal composites by electroless plating method. Integr. Ferroelectr..

[9] Kim S.S., Jeon J.H., Kee C.D., Oh I.-K. (2013). Electro-active hybrid actuators based on freeze-dried bacterial cellulose and PEDOT:PSS. Smart Mater. Struct..

[10] Terasawa N., Asaka K. (2016). High-performance PEDOT: PSS/single-walled carbon nanotube/ionic liquid actuators combining electrostatic double-layer and faradaic capacitors. Langmuir.

[11] Aabloo A., De Luca V., Di Pasquale G., Graziani S., Gugliuzzo C., Johanson U., Marino C., Pollicino A., Puglisi R. (2016). A new class of ionic electroactive polymers based on green synthesis. Sens. Actuators A Phys..

[12] Kong L., Chen W. (2014). Carbon nanotube and graphene-based bioinspired electrochemical actuators. Adv. Mater..

[13] Torop J., Aabloo A., Jager E.W.H. (2014). Novel actuators based on polypyrrole/carbide-derived carbon hybrid materials. Carbon.

[14] Inamuddin, Kashmery A.H. (2019). Polyvinylidene fluoride/sulfonated graphene oxide blend membrane coated with polypyrrole/platinum electrode for ionic polymer metal composite actuator applications. Sci. Rep..

[15] Okuzaki H., Takagi S., Hishiki F., Tanigawa R. (2014). Ionic liquid/polyurethane/PEDOT:PSS composites for electro-active polymer actuators. Sens. Actuators B Chem..

[16] Luqman M., Shaikh H.M., Anis A., Al-Zahrani S.M., Alam M.A. (2022). A Convenient and Simple Ionic Polymer-Metal Composite (IPMC) Actuator Based on a Platinum-Coated Sulfonated Poly(ether ether ketone)–Polyaniline Composite Membrane. Polymers.

[17] Zhang C., Liu Y., Li H., Liu S., Li P., Zhang H., He C. (2024). Enhanced thermoelectric properties of carbon nanotubes/polyaniline fibers through engineering doping level and orientation. Compos. Sci. Technol..

[18] Park J., Yang X., Wickramasinghe D., Sundhoro M., Orbey N., Chow K.-F., Yan M. (2020). Functionalization of pristine graphene for the synthesis of covalent graphene–polyaniline nanocomposite. RSC Adv..

[19] Al Jabri H., Devi M.G., Al-Shukaili M.A. (2023). Development of polyaniline–TiO_2_ nano composite films and its application in corrosion inhibition of oil pipelines. J. Indian Chem. Soc..

[20] Shakiba M., Abdouss M., Mazinani S., Kalaee M.R. (2023). Super-hydrophilic electrospun PAN nanofibrous membrane modified with alkaline treatment and ultrasonic-assisted PANI in-situ polymerization for highly efficient gravity-driven oil/water separation. Sep. Purif. Technol..

[21] Ikushima K., John S., Ono A., Nagamitsu S. (2010). PEDOT/PSS Bending Actuators for Autofocus Micro Lens Applications. Synth. Met..

[22] Verma A., Kumar T. (2024). Ag/Cu doped polyaniline hybrid nanocomposite-based novel gas sensor for enhanced ammonia gas sensing performance at room temperature. RSC Adv..

[23] Fu X., Wen J., Xia C., Liu Q., Zhang R., Hu S. (2023). Nafion doped polyaniline/graphene oxide composites as electrode materials for high performance flexible supercapacitors based on Nafion membrane. Mater. Des..

[24] Ryoo R., Joo S.H., Jun S. (1999). Synthesis of highly ordered carbon molecular sieves via template-mediated structural transformation. J. Phys. Chem. B.

[25] Wang D.-W., Li F., Liu M., Lu G.Q., Cheng H.-M. (2008). 3D aperiodic hierarchical porous graphitic carbon material for high-rate electrochemical capacitive energy storage. Angew. Chem. Int. Ed..

[26] Porfiri M. (2009). Influence of electrode surface roughness and steric effects on the nonlinear electromechanical behavior of ionic polymer metal composites. Phys. Rev. E.

[27] Palmre V., Pugal D., Leang K., Kim K. (2013). The effects of electrode surface morphology on the actuation performance of IPMC. Proc. SPIE.

[28] Chang L., Asaka K., Zhu Z., Wang Y., Chen H., Li D. (2014). Effects of surface roughening on the mass transport and mechanical properties of ionic polymer-metal composite. J. Appl. Phys..

[29] Li Q., Zhang Q., Sun J., Liu C., Guo J., He B., Zhou Z., Man P., Li C., Xie L. (2019). All hierarchical core-shell heterostructures as novel binder-free electrode materials for ultrahigh-energy-density wearable asymmetric super-capacitors. Adv. Sci..

[30] Park S.W., Kim S.J., Park S.H., Lee J., Kim H., Kim M.K. (2022). Recent Progress in Development and Applications of Ionic Polymer–Metal Composite. Micromachines.

[31] Sun P.-P., Zhang Y.-H., Shi H., Shi F.-N. (2022). Controllable One Step Electrochemical Synthesis of PANI Encapsulating 3d-4f Bimetal MOFs Heterostructures as Electrode Materials for High-performance Supercapacitors. Chem. Eng. J..

[32] Xiao F., Zhou H., Lin H., Li H., Zou T., Wu Y., Guo Z. (2021). A Fast Scan Cyclic Voltammetric Digital Circuit with Precise Ohmic Drop Compensation by Online Measuring Solution Resistance and Its Biosensing Application. Anal. Chim. Acta.

[33] Orazem M.E., Tribollet B. (2020). A Tutorial on Electrochemical Impedance Spectroscopy. ChemTexts.

[34] Samadpour M., Dehghani M., Parand P., Najafi M.N., Parvazian E. (2020). Photovoltaic Performance and Electrochemical Impedance Spectroscopy Analysis of CdS/CdSe-Sensitized Solar Cell Based on Surfactant-modified ZnS Treatment. Appl. Phys. A.

